# Use of multiplex PCR and real-time PCR to detect human herpes virus genome in ocular fluids of patients with uveitis

**DOI:** 10.1136/bjo.2007.133967

**Published:** 2008-04-11

**Authors:** S Sugita, N Shimizu, K Watanabe, M Mizukami, T Morio, Y Sugamoto, M Mochizuki

**Affiliations:** 1 Department of Ophthalmology & Visual Science, Medical Research Institute, Tokyo Medical and Dental University, Tokyo, Japan; 2 Department of Virology, Medical Research Institute, Tokyo Medical and Dental University, Tokyo, Japan; 3 Center for Cell Therapy, Tokyo Medical and Dental University, Tokyo, Japan

## Abstract

**Aim:**

To measure the genomic DNA of human herpes viruses (HHV) in the ocular fluids and to analyse the clinical relevance of HHV in uveitis.

**Methods:**

After informed consent was obtained, a total of 111 ocular fluid samples (68 aqueous humour and 43 vitreous fluid samples) were collected from 100 patients with uveitis. The samples were assayed for HHV-DNA (HHV1–8) by using two different polymerase chain reaction (PCR) assays, qualitative PCR (multiplex PCR) and quantitative PCR (real-time PCR).

**Results:**

In all of the patients with acute retinal necrosis (n = 16) that were tested, either the HSV1 (n = 2), HSV2 (n = 3), or VZV (n = 11) genome was detected. In all patients, high copy numbers of the viral DNA were also noted, indicating the presence of viral replication. In another 10 patients with anterior uveitis with iris atrophy, the VZV genome was detected. When using multiplex PCR, EBV-DNA was detected in 19 of 111 samples (17%). However, real-time PCR analysis of EBV-DNA indicated that there were only six of the 19 samples that had significantly high copy numbers. The cytomegalovirus (CMV) genome was detected in three patients with anterior uveitis of immunocompetent patients and in one immunocompromised CMV retinitis patient. In addition, one patient with severe unilateral panuveitis had a high copy number of HHV6-DNA. There was no HHV7- or HHV8-DNA detected in any of the samples.

**Conclusions:**

A qualitative multiplex PCR is useful in the screening of viral infections. However, the clinical relevance of the virus infection needs to be evaluated by quantitative real-time PCR.

Human herpes virus (HHV) affects various ocular tissues and is known to cause anterior and/or posterior uveitis, which is characterised by mutton-fat keratic precipitates (KPs), ocular hypertension, iris atrophy, vitreous opacity, and necrotic retinitis. Using polymerase chain reaction (PCR), previous studies have demonstrated the presence of genomic DNA for HHV in the aqueous humour and vitreous fluids in patients with herpetic uveitis, including herpetic keratouveitis, herpes zoster ophthalmicus, zoster sine herpete, acute retinal necrosis, and cytomegalovirus retinitis.^1^^–^7 With recent advances in molecular biology, use of real-time PCR now makes it possible for quantitative measurements of the viral load associated with herpes virus diseases in the eye.^5^ ^6^ In addition, multiplex qualitative PCR has the advantage of combining several different primer pairs in the same amplification reaction with the net result of producing different specific virus-amplicons in ocular infectious diseases.^7^ Therefore, multiplex PCR can be used to detect the presence of viruses within samples.

In this study, we collected ocular samples from various uveitis patients and then tried to detect the HHV genome when using combinations of two PCR systems: (1) multiplex qualitative PCR and (2) real-time quantitative PCR.

## MATERIAL AND METHODS

### Subjects

Samples of aqueous humour (n = 68) and vitreous fluid (n = 43) were collected from 100 patients with uveitis and ocular lymphoma. Underlying pathology comprised herpetic keratouveitis (n = 7), herpetic anterior uveitis/iridocyclitis (n = 16), acute retinal necrosis (ARN; n = 16), cytomegalovirus (CMV) retinitis (n = 1), human T lymphotropic virus type 1 (HTLV-1) uveitis (n = 1), ocular toxoplasmosis (n = 2), scleritis (n = 3), ocular sarcoidosis (n = 7), Vogt–Koyanagi–Harada (VKH) disease (n = 2), Behçet disease (n = 2), idiopathic uveitis (n = 26) and intraocular lymphoma (n = 12). At the time of sampling, uveitis patients displayed active intraocular inflammation.

An aliquot of 0.1 ml of the aqueous humour was aspirated with a 30 G needle. In patients with uveitis who were undergoing vitreous surgery, non-diluted vitreous fluid samples were collected from the patients during surgery (diagnostic pars-plana vitrectomy). The samples used in this study were collected between 1999 and 2007.

### Polymerase chain reaction

**Figure 1:**
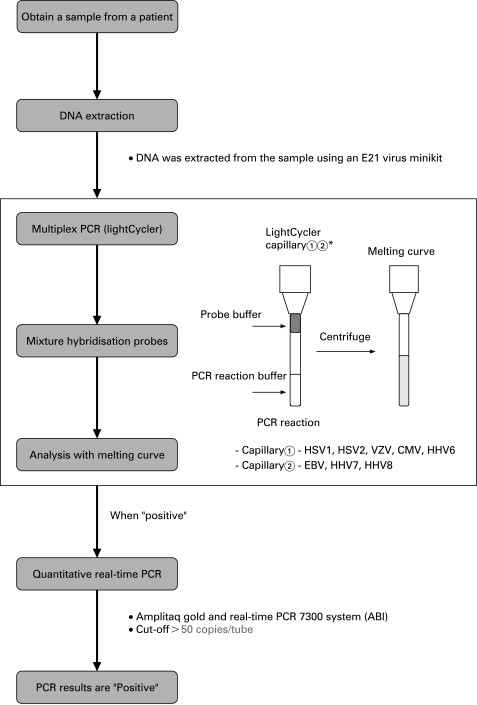
Use of multiplex PCR and real-time PCR for the analysis of human herpes virus family genomic DNA in ocular fluids of patients with uveitis. We performed independent PCR methods to detect herpes viruses, using both a qualitative multiplex PCR and a quantitative real-time PCR. After DNA extraction from each of the samples, multiplex PCR was performed to screen from HHV1 to HHV8 using two LightCycler capillaries. When a “positive” result was observed, real-time PCR was performed to measure the viral load. When more than 50 copies/tube (5×10^3^/ml) were observed, the value was considered to be significant. CMV, cytomegalovirus; EBV, Epstein–Barr virus; HHV, human herpes virus; HSV, herpes simplex virus; VZV, Varicella-zoster virus.

Genomic DNA of HHV in the aqueous humour and vitreous fluids was measured through the use of two independent PCR assays: (1) a qualitative multiplex PCR and (2) a quantitative real-time PCR. The result analysis for the PCR is shown in fig 1.

DNA was extracted from samples using an E21 virus minikit (Qiagen, Valencia, CA) installed on a Robotic workstation for automated purification of nucleic acids (BioRobot E21, Qiagen). The multiplex PCR was designed to qualitatively measure genomic DNA of eight human herpes viruses, that is, herpes simplex virus type 1 (HSV-1), type 2 (HSV-2), Varicella-zoster virus (VZV), Epstein–Barr virus (EBV), cytomegalovirus (CMV), human herpes virus type 6 (HHV6), type 7 (HHV7) and type 8 (HHV8). The PCR was performed using a LightCycler (Roche, Switzerland). Primers and probes of HHV1–8 and the PCR conditions have been described previously.^8^ Specific primers for the virus were used with Accuprime Taq (Invitrogen, Carlsbad, CA). Products were subjected to 40 cycles of PCR amplification. Hybridisation probes were then mixed with the PCR products. Subsequently, real-time PCR was performed only for the human herpes virus, with the genomic DNA detected by multiplex PCR (fig 1). The real-time PCR was performed using Amplitaq Gold and the Real-Time PCR 7300 system (ABI, Foster City, CA). The sequence of the HHV1–8 primers and probes are shown in table 1. The primers of the viruses and the PCR conditions have been described in previous reports.^9^^–^13 Our research group has also previously reported the primers of the sequences for VZV.^14^ All of the products obtained were subjected to 45 cycles of PCR amplification. The value of viral copy number in the sample was considered to be significant, when more than 50 copies/tube (5×10^3^/ml) were observed.

**Table 1:** Sequence for primers and probes in human herpes viruses (HHV) using real-time PCR

Herpes virus	Sequence for primers and probes	Amplification
HSV1 and 2	HSV-F: CGCATCAAGACCACCTCCTC	gB
	HSV-R: GCTCGCACCACGCGA	
	HSV1-P: JOE-TGGCAACGCGGCCCAAC-TAMRA	
	HSV2-P: FAM-CGGCGATGCGCCCCAG-TAMRA	
VZV	VZV-F: AACTTTTACATCCAGCCTGGCG	ORF29
	VZV-R: GAAAACCCAAACCGTTCTCGAG	
	VZV-P: FAM-TGTCTTTCACGGAGGCAAACACGT-TAMRA	
EBV	EBV-F: CGGAAGCCCTCTGGACTTC	BALF5
	EBV-R: CCCTGTTTATCCGATGGAATG	
	EBV-P: FAM-TGTACACGCACGAGAAATGCGCC-TAMRA	
CMV	CMV-F: CATGAAGGTCTTTGCCCAGTAC	IE-1
	CMV-R: GGCCAAAGTGTAGGCTACAATAG	
	CMV-P: FAM-TGGCCCGTAGGTCATCCACACTAGG-TAMRA	
HHV6	HHV6-F: GACAATCACATGCCTGGATAATG	U65-U66
	HHV6-R: TGTAAGCGTGTGGTAATGTACTAA	
	HHV6-P: FAM-AGCAGCTGGCGAAAAGTGCTGTGC-TAMRA	
HHV7	HHV7-F: CGGAAGTCACTGGAGTAATGACAA	U37
	HHV7-R: CCAATCCTTCCGAAACCGAT	
	HHV7-P: FAM-CTCGCAGATTGCTTGTTGGCCATG-TAMRA	
HHV8	HHV8-F: CCTCTGGTCCCCATTCATTG	ORF65
	HHV8-R: CGTTTCCGTCGTGGATGAG	
	HHV8-P: FAM-CCGGCGTCAGACATTCTCACAACC-TAMRA	

The real-time herpes simplex virus (HSV) PCR is a multiplexing PCR that can detect both HSV1 and HSV2 DNA in the same reaction. The optimised gB primer pairs amplify both HSV1 and 2 with equal efficiency, with the two type-specific probes labelled with different fluorescent dyes. HSV1 probe is labelled with JOE at the 5′-end and with TAMRA at the 3′-end. HSV2 probe is labelled with FAM at the 5′-end and with TAMRA at the 3′-end.

CMV, cytomegalovirus; EBV, Epstein–Barr virus; VZV, Varicella-zoster virus.

## RESULTS

Our initial PCR results indicated HHV positivity in the ocular fluids of uveitis patients. As shown in table 2, multiplex PCR detected seven patients with HSV1-DNA while real-time PCR found that all seven of these patients also had a high HSV1 viral load. In addition, HSV2-DNA was detected in three patients, with all of these patients having a high viral load. In 29 patients, VZV-DNA was detected, but only 21 patients (72%) had a high viral load. EBV was detected in 19 patients, but only six out of the 19 cases were positive (32%). CMV-DNA was detected in six patients, with four out of the six cases (67%) found to be positive by the real-time PCR. HHV6-DNA was detected in only one patient by both of the PCR methods. There were no patients for which HHV7 and HHV8 were detected. Overall, there were 65 multiplex PCR positive patients and 42 real-time PCR positive patients (table 2). Clinically, we decided that only if HHV-DNA could be detected in a sample (aqueous humour and/or vitreous) by both PCR methods would the patient then be considered to be positive. If a patient was found to be positive by only one of the PCR methods, for example, positive by multiplex qualitative PCR and negative (<50 copies/tube) by real-time quantitative PCR, we did not take this as PCR-positive (fig 1).

**Table 2:** Human herpes virus-PCR positivity in ocular fluids of 100 patients with uveitis

Herpes virus	Multiplex PCR	Real-time PCR
HSV1	7/100 (7%)	7/7 (100%)
HSV2	3/100 (3%)	3/3 (100%)
VZV	29/100 (29%)	21/29 (72%)
EBV	19/100 (19%)	6/19 (32%)
CMV	6/100 (6%)	4/6 (67%)
HHV6	1/100 (1%)	1/1 (100%)
HHV7	0/100 (0%)	–
HHV8	0/100 (0%)	–
Total	65/100 (65%)	42/65 (65%)

Qualitative multiplex PCR was performed in order to screen for and detect human herpes virus (HHV) genomic DNA, HHV1–HHV8. When the genomic DNA was detected by the multiplex PCR (n = 65), real-time PCR was then performed only for the HHV.

CMV, cytomegalovirus; EBV, Epstein–Barr virus; HSV, herpes simplex virus; VZV, Varicella-zoster virus.

Subsequently, we analysed the results for each the viruses, from HHV1 to HHV8. The summary of the results is shown in table 3. HSV1 was detected in two cases of keratouveitis, and in three cases of anterior uveitis. These patients had mutton-fat KPs, ocular hypertension and anterior chamber cells. HSV1 was also detected in two cases of acute retinal necrosis (ARN). HSV2 was detected in three cases of ARN. VZV was detected in 10 cases of herpetic anterior uveitis and in 11 cases of ARN. During the time after the initial onset of anterior uveitis, iris atrophy developed in these patients. Higher viral load in the aqueous humour was well correlated with tissue damage, such as iris atrophy.^14^ In addition, as we have reported previously, real-time PCR of the ocular fluids from ARN patients (n = 16) indicated high viral loads of VZV (n = 11, 69%), HSV1 (n = 2, 12%), and HSV2 (n = 3, 19%).^8^

**Table 3:** PCR results for each herpes virus genome in patients with uveitis

Herpes virus	Clinical diagnosis	PCR-positive*/total no of patients
HSV1	Herpetic keratouveitis	2/7†
	Herpetic anterior uveitis	3/16
	Acute retinal necrosis	2/16
	Others	0/61
HSV2	Acute retinal necrosis	3/16
	Others	0/84
VZV	Herpetic anterior uveitis	10/16
	Acute retinal necrosis	11/16
	Others	0/68
EBV	Idiopathic uveitis	1/26
	Herpetic anterior uveitis (VZV)	2/16
	Acute retinal necrosis (VZV)	2/16
	Intraocular lymphoma	1/12
	Others	0/30
CMV	Herpetic anterior uveitis	3/16
	Cytomegalovirus retinitis	1/1
	Others	0/83
HHV6	Idiopathic uveitis	1/26
	Others	0/74
HHV7	–	0/100
HHV8	–	0/100

*Detection of HHV-DNA by both multiplex PCR and real-time PCR.

†In the seven patients with keratouveitis, our PCR system detected HSV1-DNA in two patients.

CMV, cytomegalovirus; EBV, Epstein–Barr virus; HHV, human herpes virus; HSV, herpes simplex virus; VZV, Varicella-zoster virus.

EBV was detected in only one case of idiopathic uveitis. This patient had acute anterior uveitis with hypopyon and was HLA-B27 negative. Therefore, as previously reported, we diagnosed EBV-related acute anterior uveitis.^15^ EBV was also detected in two cases of VZV-associated anterior uveitis and in two cases of VZV-ARN. This suggests that these patients have a high copy number of VZV, as well as EBV in their ocular fluids. EBV was also detected in one case of ocular B-cell lymphoma.

**Figure 2:**
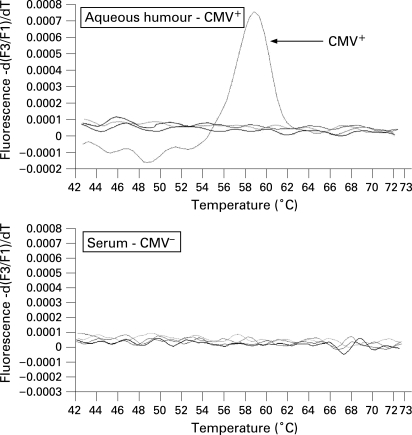
Results for a multiplex PCR in a patient with anterior uveitis. At 58°C, a significant positive curve was seen, indicating the detection of cytomegalovirus (CMV) genomic DNA in the aqueous humour. The other herpes viruses, such as herpes simplex virus (HSV) 1, HSV2, Varicella-zoster virus, Epstein–Barr virus, human herpes virus (HHV6), HHV7 and HHV8, were found to be negative in this particular sample. In addition, CMV-DNA was not detected in the patient’s serum.

CMV was detected in a case of cytomegalovirus retinitis and in three cases of CMV-associated anterior uveitis. Representative results from the multiplex qualitative PCR can be seen in fig 2. CMV-DNA was detected in the aqueous humour, and quantitative real-time PCR revealed there were 2.3×10^5^ copies/mL of CMV-DNA in the specimen. As we previously reported, in the affected eye there were whitish small-size mutton-fat KPs along with mild inflammation in the anterior chamber.^16^ During the 8 years this particular patient was followed, he had been considered to have a case of Posner–Schlossman syndrome. This patient had no retinitis, and additionally he was not found to be immunocompromised.

A high copy number of HHV6-DNA was detected in only one patient with severe unilateral panuveitis. In this patient, multiple retinal exudates, vitreous opacity, along with a whitish mass lesion were observed in the affected eye. We reported this case as HHV6-associated panuveitis.^17^ In the current study, HHV7- or HHV8-DNA was not detected in any of the patients (table 3).

## DISCUSSION

Human herpes viruses (HHV) can widely affect the eye and be expressed in ocular tissues or excreted in ocular fluids. Previously, the diagnosis of intraocular HHV infection was made by measuring local production of specific anti-virus antibodies—for example, using the Goldmann–Witmer coefficient. Recently, diagnosis has also been performed through the detection of the virus genome by PCR. Cell-free herpes virus DNA has been detected in the aqueous humour and vitreous fluids of patients with uveitis.^1^^–^7 In the current study, we showed that intraocular HHV-DNA was detectable over a wide range of HHV-associated uveitis when analysis was performed using the two PCR methods. Thus, the current PCR system may be a valuable tool in the diagnosis of infectious uveitis. In addition, with the use of these examinations, this allows non-herpetic uveitis patients to be excluded.

When faced with a clinical situation that suggests a differential diagnosis of HHV1–8, the multiplex PCR assay can provide a rapid and reliable diagnosis, even when only small sample amounts are available for examination in the ocular microbiology laboratory. The majority of the viruses associated with eye diseases are related to the herpes virus group. Therefore, the last decade has seen several studies concluding that herpes virus PCR-based laboratory investigations are valuable tools in the diagnosis of viral diseases of the eye. The advantages of developing the multiplex PCR assay are obvious, with several of them reported as being useful in the detection of herpes viruses when various combinations are employed.^7^ ^18^ ^19^ In the current study, we were able to rapidly screen for the detection of the virus genome of all eight types of human herpes viruses by using several different primer pairs. When positive results were noted, we then used real-time quantitative PCR to examine the viral load using different primer pairs. This allowed us to confirm our positive results through the use of two PCR combinations.

It is important to discuss the significance of the high viral load of HHV in these patients. The finding of high viral loads in the ocular fluids indicates that virus replication takes place in the eye, suggesting a direct pathogenic role in intraocular inflammation. In the current study, some patients had previously been on systemic or topical corticosteroids over long periods of times before we collected the ocular samples. Therefore, we have to consider that long-term usage of topical and/or systemic steroids might be responsible for the creation of a steroid reservoir that could lead to a localised immunosuppressed state, thereby resulting in HHV replication. In order to be able to avoid the irreversible tissue damage and visual impairment caused by the viral infection, early treatment with anti-viral agents is clinically important, and this can be achieved if there is a rapid and accurate diagnosis of the viral infection in ocular tissues by PCR.

In summary, the HHV family-DNA was detected by multiplex PCR in the ocular fluids of patients with various types of uveitis. Among the positive samples that were identified through the use of qualitative PCR, many of these samples showed significantly high copy numbers of HHV-DNA, when examined by real-time PCR. However, it should be noted that levels of the viral load for some of the intraocular samples could not be detected, for example, as was seen for the real-time PCR measurement of EBV. Thus, a qualitative multiplex PCR might be a useful method for screening viral infections, and furthermore, quantitative real-time PCR might make it possible to evaluate the clinical relevance of virus infections.

## References

[1] Ohashi Y Yamamoto S Nishida K Demonstration of herpes simplex virus DNA in idiopathic corneal endotheliopathy. Am J Ophthalmol 1991;112:419–23 1656756 10.1016/s0002-9394(14)76251-8

[2] Yamamoto S Tada R Shimomura Y Detecting varicella-zoster virus DNA in iridocyclitis using polymerase chain reaction. Arch Ophthalmol 1995;113:1358–9 7487588 10.1001/archopht.1995.01100110018009

[3] Nakamura N Tanabe M Yamada Y Zoster sine herpete with bilateral ocular involvement. Am J Ophthalmol 2000;129:809–10 10926998 10.1016/s0002-9394(00)00404-9

[4] Koizumi N Yamasaki K Kawasaki S Cytomegalovirus in aqueous humor from an eye with corneal endotheliitis. Am J Ophthalmol 2006;141:564–5 16490509 10.1016/j.ajo.2005.09.021

[5] Arimura E Deai T Maruyama K Herpes simplex virus-2 quantification by real-time polymerase chain reaction in acute retinal necrosis. Jpn J Ophthalmol 2005;49:64–5 15692781 10.1007/s10384-004-0145-0

[6] Asano S Yoshikawa T Kimura H Monitoring herpesvirus DNA in three cases of acute retinal necrosis by real-time PCR. J Clin Virol 2004;29:206–9 15002491 10.1016/s1386-6532(03)00162-8

[7] Chichili GR Athmanathan S Farhatullah S Multiplex polymerase chain reaction for the detection of herpes simplex virus, varicella-zoster virus and cytomegalovirus in ocular specimens. Curr Eye Res 2003;27:85–90 14632159 10.1076/ceyr.27.2.85.15947

[8] Sugita S Iwanaga Y Kawaguchi T Detection of herpesviruses genome by multiplex PCR and real-time PCR in ocular fluids of patients with acute retinal necrosis [in Japanese]. Nippon Ganka Gakkai Zasshi 2008;112:30–8 18240601

[9] Corey L Huang ML Selke S Differentiation of herpes simplex virus types 1 and 2 in clinical samples by a real-time taqman PCR assay. J Med Virol 2005;76:350–5 15902702 10.1002/jmv.20365

[10] Kimura H Morita M Yabuta Y Quantitative analysis of Epstein–Barr virus load by using a real-time PCR assay. J Clin Microbiol 1999;37:132–6 9854077 10.1128/jcm.37.1.132-136.1999 PMC84187

[11] Gautheret-Dejean A Manichanh C Thien-Ah-Koon F Development of a real-time polymerase chain reaction assay for the diagnosis of human herpesvirus-6 infection and application to bone marrow transplant patients. J Virol Meth 2002;100:27–35 10.1016/s0166-0934(01)00390-1 11742650

[12] Hara S Kimura H Hoshino Y Detection of herpesvirus DNA in the serum of immunocompetent children. Microbiol Immnol 2002;46:177–80 10.1111/j.1348-0421.2002.tb02683.x 12008926

[13] Polstra AM van den Burg R Goudsmit J Human herpesvirus 8 load in matched serum and plasma samples of patients with AIDS-associated Kaposi’s sarcoma. J Clin Microbiol 2003;41:5488–91 14662929 10.1128/JCM.41.12.5488-5491.2003 PMC308989

[14] Kido S Sugita S Horie S Association of varicella-zoster virus (VZV) load in the aqueous humor with clinical manifestations of anterior uveitis in herpes zoster ophthalmicus and zoster sine herpete. Br J Ophthalmol 2008;92:505–8 18245272 10.1136/bjo.2007.125773

[15] Takahashi H Sugita S Shimizu N A high viral load of Epstein–Barr virus (EBV) DNA in ocular fluids in a HLA-B27 negative acute anterior uveitis patient with psoriasis. Jpn J Ophthalmol. In press. 10.1007/s10384-007-0508-4 18626741

[16] Kawaguchi T Sugita S Shimizu N Kinetics of aqueous flare, intraocular pressure and virus-DNA copies in a patient with cytomegalovirus iridocyclitis without retinitis. Inter Ophthalmol 2007;27:383–6 10.1007/s10792-007-9090-5 17522780

[17] Sugita S Shimizu N Kawaguchi T Identification of human herpesvirus 6 in a patient with severe unilateral panuveitis. Arch Ophthalmol 2007;125:1426–7 17923557 10.1001/archopht.125.10.1426

[18] Elnifro EM Cooper RJ Klapper PE Multiplex polymerase chain reaction for diagnosis of viral and chlamydial keratoconjunctivitis. Invest Ophthalmol Vis Sci 2000;41:1818–22 10845603

[19] Druce J Catton M Chibo D Utility of a multiplex PCR assay for detecting herpesvirus DNA in clinical samples. J Clin Microbiol 2002;40:1728–32 11980951 10.1128/JCM.40.5.1728-1732.2002 PMC130932

